# The silent risk: Occult Hepatitis B virus detected in Cornea donors

**DOI:** 10.1016/j.htct.2026.108549

**Published:** 2026-10-16

**Authors:** Guilherme Louzada Silva Meira, Josiane Iole França Lopes, Marinna Garcia Azevedo Repossi, Alexandra Martins Soares, Luiz Amorim Filho

**Affiliations:** HEMORIO, Instituto Estadual de Hematologia Arthur de Siqueira Cavalcanti, Rio de Janeiro, Brazil

## Introduction

Approximately 254 million people worldwide are chronically infected with the hepatitis B virus (HBV), which is responsible for 1.1 million deaths annually [1]. HBV infection is associated with a broad spectrum of clinical manifestations, ranging from acute or fulminant hepatitis to various chronic forms, including asymptomatic carriage, chronic hepatitis, cirrhosis, and hepatocellular carcinoma (HCC) [2]. In 2022, an estimated 1,038,564 people in Brazil were infected with HBV; however, only 23.1% had a laboratory-confirmed diagnosis [1]. The importance of monitoring HBV transmission risk during blood transfusions gained prominence in Brazil across all blood centers in the country following the implementation of the national Nucleic Acid Test (NAT) platform for human immunodeficiency virus (HIV), hepatitis C virus (HCV), and HBV detection, developed by Bio-Manguinhos (FIOCRUZ, Rio de Janeiro, Brazil) and the Ministry of Health [3]. Conversely, under current Brazilian eye banking guidelines, NAT is recommended rather than mandatory [4].

### Case presentation and discussion

Nucleic acid testing (NAT) was performed on blood samples retrieved from ocular and tissue banks in Rio de Janeiro and Espírito Santo, Brazil. Among the assays performed, HBV DNA with a cycle threshold (CT) of 33 was detected in a blood sample from a 58-year-old male cornea donor from the eye bank of Espírito Santo State, Brazil. Although exact viral load quantification was not performed, this case represents an occult HBV infection (OBI), as the cornea donor was HBsAg non-reactive but reactive for total anti-HBc and anti-HBs (225.26 mIU/mL). OBI is defined as the presence of HBV DNA in blood or liver tissues in the absence of detectable HBsAg, with or without the presence of anti-HBV antibodies [5]. While OBIs are typically associated with low viral loads, which reduces the risk of transmission, this risk may be heightened in the context of corneal transplantation. The donor's high Anti-HBs titer highlights a hidden risk: these protective antibodies circulate in the donor's blood and are not transferred with the avascular cornea. Consequently, the recipient receives the tissue carrying the viral DNA, but without the donor's humoral protection. Because corneal transplant recipients are routinely subjected to immunosuppressive eye drops (e.g., topical corticosteroids), their local immune defense is weakened, creating an environment where viral reactivation can indeed occur. The risk of HBV transmission via corneal transplantation, while low, has been documented [6], and HBV has been demonstrably detected in corneal tissues and ocular fluids [[7], [8], [9]].

To further contextualize this issue, it is important to highlight the contrasting regulatory frameworks in Brazil regarding anti-HBc reactivity. In the context of blood donation, a reactive anti-HBc test renders the donor ineligible and mandates the discard of the collected blood unit [10]. Conversely, in deceased organ and tissue donation, an anti-HBc reactive donor may be accepted under specific circumstances: anti-HBc positive and HBsAg negative donors can be accepted if the transplant candidate possesses any HBV serological marker, or if a seronegative recipient receives antiviral prophylaxis or undergoes strict post-transplant infection surveillance. For eye banks, where NAT is not currently mandated [4], we propose that NAT could serve as a valuable tool to mitigate transmission risks and guide the clinical decisions of the transplant team, especially when evaluating donors who are anti-HBc reactive but HBsAg non-reactive.

## Conflicts of interest

The authors declare that they have no known competing financial interests or personal relationships that could have appeared to influence the work reported in this article.

## Data Availability

The data supporting the findings of this study were generated from routine nucleic acid testing (NAT) performed on donor screening samples from eye and tissue banks in Brazil. Due to legal and ethical restrictions related to donor confidentiality and applicable Brazilian data protection requirements, the underlying individual-level data are not publicly available. De-identified data may be made available from the corresponding author upon reasonable request and subject to institutional approval.
